# miR-141 mediates recovery from acute kidney injury

**DOI:** 10.1038/s41598-021-94984-x

**Published:** 2021-08-13

**Authors:** Lucy J. Newbury, Kate Simpson, Usman Khalid, Imogen John, Lluís Bailach de Rivera, Yueh-An Lu, Melisa Lopez-Anton, William J. Watkins, Robert H. Jenkins, Donald J. Fraser, Timothy Bowen

**Affiliations:** 1 Wales Kidney Research Unit, Division of Infection and Immunity, School of Medicine, College of Biomedical and Life Sciences, Cardiff University, Heath Park, Cardiff, CF14 4XN UK; 2 Cardiff Institute of Tissue Engineering and Repair, Museum Place, Cardiff, CF10 3BG UK; 3 Division of Nephrology, Kidney Research Center, Linkou Chang Gung Memorial Hospital, Linkou, Taiwan; 4 Division of Infection and Immunity, School of Medicine, College of Biomedical and Life Sciences, Cardiff University, Heath Park, Cardiff, CF14 4XN UK

**Keywords:** Biomarkers, Nephrology

## Abstract

Acute kidney injury (AKI) is a global clinical problem characterised by a sudden decline in renal function and mortality as high as 60%. Current AKI biomarkers have limited ability to classify disease progression and identify underlying pathological mechanisms. Here we hypothesised that alterations in urinary microRNA profiles could predict AKI recovery/nonrecovery after 90 days, and that injury-specific changes would signify microRNA mediators of AKI pathology. Comparison of urinary microRNA profiles from AKI patients with controls detected significant injury-specific increases in miR-21, miR-126 and miR-141 (p < 0.05) and decreases in miR-192 (p < 0.001) and miR-204 (p < 0.05). Expression of miR-141 increased in renal proximal tubular epithelial cells (PTECs) under oxidative stress in vitro and unilateral ischaemic reperfusion injury in vivo*.* Forced miR-141 expression in the presence of H_2_O_2_ increased PTEC death and decreased cell viability. Of nine messenger RNA targets with two or more miR-141 3’-untranslated region binding sites, we confirmed protein tyrosine phosphatase receptor type G (PTPRG) as a direct miR-141 target in PTECs. PTPRG-specific siRNA knockdown under oxidative stress increased PTEC death and decreased cell viability. In conclusion, we detected significant alterations in five urinary microRNAs following AKI, and identified proximal tubular cell PTPRG as a putative novel therapeutic target.

## Introduction

Acute kidney injury (AKI) is a global clinical problem that is characterised by abrupt loss of renal function^1^ and is a current UK healthcare focus due to its cost implications^2,3^. Indeed, recent data suggest that a quarter of patients with SARS-Cov-2 infection develop AKI^4^. Despite the importance of AKI in determining patient outcomes following hospital admission, diagnosis and treatment remain challenging^2,3^. Current clinical guidelines use serum creatinine and urine output to define AKI^1,2,5^, but these biomarkers lack sensitivity and specificity, changing meaningfully only after significant kidney injury has occurred. These markers are also unable to predict clinically relevant outcomes such as recovery from AKI, or to identify underlying disease aetiologies.

Identifying effective AKI biomarkers is challenging, since the initial pathological insult may be caused by a variety of factors^1^. Furthermore, there may be singular or sequential AKI episodes, which complicates identification and prognostic testing strategies. To date, the majority of biomarker research has focused on tests for earlier AKI detection. The US Food and Drug Administration has recently approved Nephrocheck, a detection test for increased urinary tissue inhibitor of metalloproteinases 2 and insulin-like growth factor binding protein 7^6–8^. This assay predicts AKI 12 h earlier than serum creatinine, improving AKI management in specific at-risk subgroups^7,8^. However, for many patients earlier detection is not realistic prior to AKI presentation, and pre-emptive testing is therefore not possible. Currently there are no approved biomarkers for the prediction of 90 day outcomes in AKI patients.

MicroRNAs (miRNAs) are ubiquitously expressed, short noncoding RNAs that show significant promise as kidney disease biomarkers. We have developed techniques for sensitive, robust detection of urinary miRNAs and demonstrated their stability via association with extracellular vesicles and/or argonaute 2 protein^9–11^. By these means, we have identified urinary miRNA biomarkers of advanced diabetic kidney disease and delayed graft function following renal transplantation^12,13^.

In addition to their roles as biomarkers, miRNAs regulate the expression of at least 60% of protein coding genes^14–16^, mediating cellular functions including proliferation and apoptosis^17^, as well as key stages in AKI progression^18,19^. These functional roles suggest that miRNAs might provide valuable insights into the molecular mechanisms of this complex and heterogeneous disease, highlighting novel pathways for clinical intervention.

In this study we hypothesised that alterations in urinary miRNA profiles could predict AKI recovery/nonrecovery after 90 days, and that injury-specific changes to renal proximal tubular epithelial cells (PTECs) would signify miRNA mediators of AKI pathology. To test our hypothesis we compared miRNAs in urine samples from stage 3 AKI patients collected at the point of AKI and correlated these findings with their 90 day recovery/nonrecovery outcomes to identify biomarkers. We then manipulated expression of selected miRNAs in injury models to investigate underlying AKI mechanisms.

## Results

### Detection of differentially expressed urinary miRNAs in AKI patients

Candidate AKI biomarkers were identified by unbiased profiling of 377 miRNAs in pooled urine samples from recovered (n = 6) and nonrecovered (n = 5) AKI patients together with archived control data (n = 20)^12^. Samples were pooled before profiling. While this approach has the potential limitation of not detecting all variations in miRNA expression, it minimises the contribution of subject-to-subject variation and makes substantive features easier to find, thereby identifying biomarkers common across individuals^12^.

**Figure 1:**
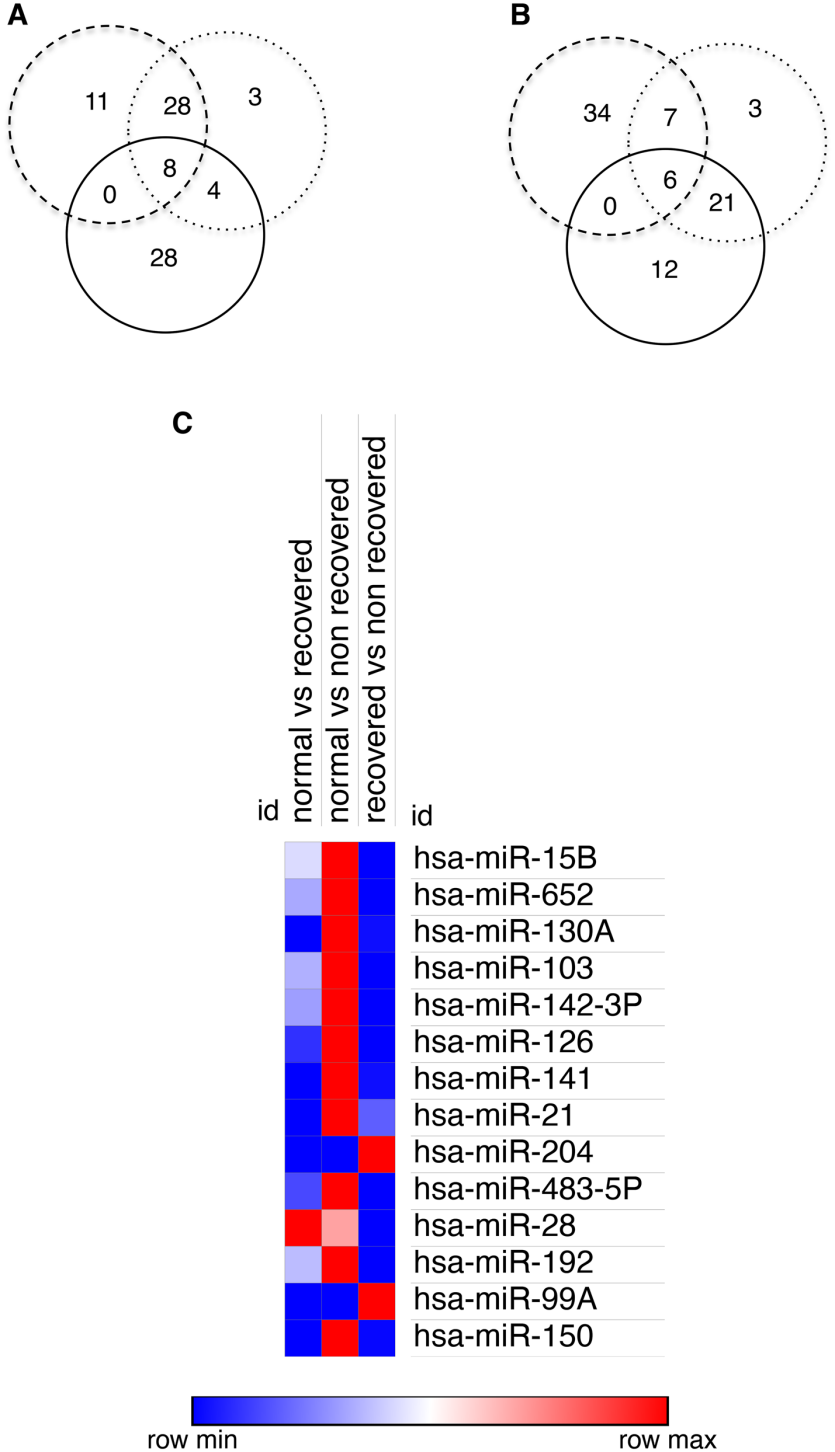
Comparison of expression profiles of 377 urinary miRNAs in pooled urine samples from recovered and nonrecovered AKI patients. (**A**,**B**) Using TaqMan Low Density Array (TLDA) Human microRNA Card A with global normalisation, urinary miRNAs from recovered AKI patients (n = 6), nonrecovered AKI patients (n = 5) and control subjects (n = 20)^11^ were analysed. Venn diagrams depicting miRNAs with ≥ twofold increased (**A**) and decreased (**B**) detection when comparing recovered AKI with controls (dashed circle), non-recovered AKI with controls (dotted circle) and non-recovered AKI with recovered AKI (solid circle). (**C**) Heatmap showing changes between recovered, non-recovered and controls with a ≥ twofold change in all three comparisons. The heatmap was generated using Morpheus software from the Broad Institute (Morpheus, https://software.broadinstitute.org/morpheus).

Data for miRNAs with a ≥ twofold change comparing recovered AKI patients with controls (47 miRNAs increased, 47 decreased), nonrecovered patients with control (43, 37), and ≥ 1.5 fold change between recovered and nonrecovered patients (40, 39) were visualised as Venn diagrams (Fig. 1A,B). The combined data highlighted 8 miRNAs that increased in all three comparisons: miR-15b, miR-21, miR-103, miR-126, miR-130a, miR-141, miR-142-3p and miR-652; and 6 that decreased: miR-28-3p, miR-99a, miR-150, miR-192, miR-204 and miR-483-5p (Fig. 1A,B). Detection data for these 14 miRNAs are displayed as a heat map in Fig. 1C.

### Complete cohort analysis confirms elevated urinary miR-21, miR-126 and miR-141, together with decreased urinary miR-192 and miR-204 in AKI patients

**Figure 2:**
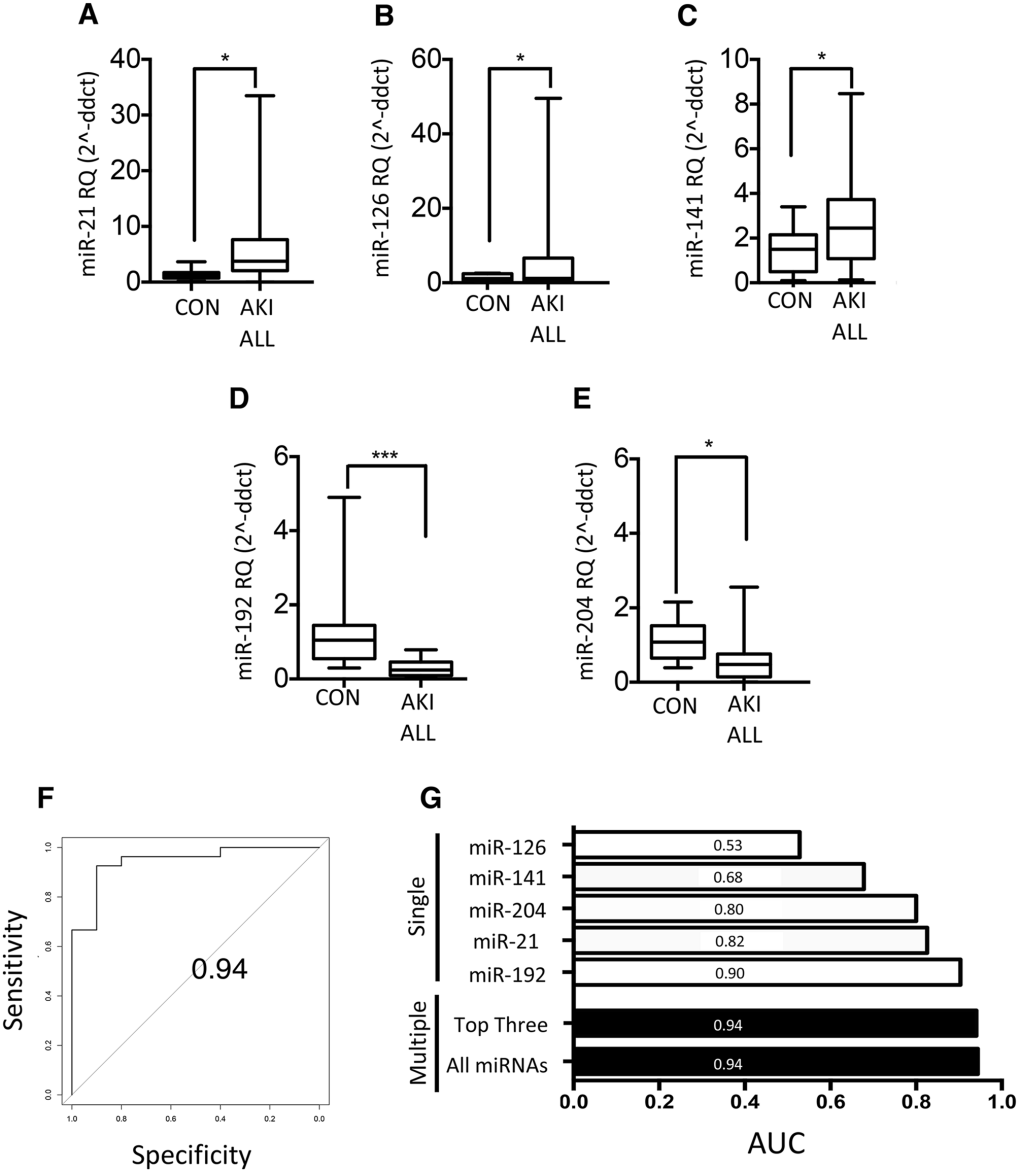
RT-qPCR detection of candidate miRNA biomarkers in AKI patient urine samples and control subjects. (**A**-**E**) Significant differences were observed between AKI patients (n = 29) and controls (n = 10) for (A) miR-21 (sevenfold increase), (**B**) miR-126 (fourfold increase), (**C**) miR-141 (twofold increase), (**D**) miR-192 (50% decrease) and (**E**) miR-204 (50% decrease). (**F**) Receiver operating characteristic (ROC) curve comparing all AKI patients with controls for all five miRNAs gave an area under the curve (AUC) = 0.94. (**G**) Bar graphs of AUCs for single and multiple miRNA comparisons between AKI patients compared to controls. Statistical analysis comparing two groups was carried out by Mann–Whitney U test. Data were normalised to endogenous control miR-191 and are presented as median ± range; *p < 0.05, ***p < 0.001. CON: control; AKI ALL: all AKI patients; Top Three: miR-192, miR-21 and miR-204.

To test the above findings, 5 candidate miRNAs were selected on the basis of relative abundance and previously reported findings, and were quantified in each urine sample from the complete stage 3 AKI cohort (n = 29; Table 1). Full notation for these miRNAs (e.g. hsa-miR-141-3p) is provided in the Methods section, their abbreviated symbols (e.g. miR-141) are used elsewhere. Significant differences were seen between patients and controls for miR-21 (sevenfold increase, p < 0.05), miR-126 (fourfold increase, p < 0.05), miR-141 twofold increase, p < 0.05), miR-192 (50% decrease, p < 0.001) and miR-204 (50% decrease, p < 0.05) (Fig. 2A-E, respectively). RT-qPCR data for all five miRNAs were used to compare AKI patients to controls in the combined receiver operating characteristic (ROC) curve analysis shown in Fig. 2F, giving an area under the curve (AUC) of 0.94. As shown in the corresponding bar graph (Fig. 2G) combined analyses performed better than comparisons of individual miRNA data.

**Table 1:** Demographic and clinical parameters of patients recruited from Wales Kidney Research Tissue Bank, University Hospital of Wales, Cardiff.

Demographics of cohort	All patients
Age (mean, ± S.D)	62.4 (19.9)
Men n (%)	17 (56.7)
**Creatinine (μmol/L) (mean, ± S.D)**	
Baseline	100.8 (31.1)
When AKI identified	479.4 (322.3)
at D0 (study entry)	493.6 (283.4)
at D90	168.4 (106.4)
No. of days between development of AKI and D0 (mean, ± S.D)	8.5 (10.2)
**Recovery status at D90**	
Nonrecovered (%)	8 (28)
Unknown (%)	3 (10)
**AKI cause**	
Pre-renal (%)	14 (47)
Inflammatory tubular (%)	10 (34)
Glomerular (%)	4 (14)
Obstruction (%)	2 (7)
**PCR (mg/mmol)**	
<15 (%)	0 (0)
15–49 n (%)	5 (17.2)
50–99 n (%)	9 (31.0)
100–299 n (%)	6 (20.7)
>300 n (%)	9 (31.0)

### Elevated urinary miR-141 and decreased miR-192 are associated with AKI nonrecovery at 90 days

An unmet clinical need for AKI biomarkers persists since existing markers cannot predict 90 day recovery or nonrecovery following the initial AKI insult. This study followed a cohort of patients with KDIGO Stage 3 AKI on study entry, whose 90 day outcomes were then determined post study. At 90 days, recovery was defined as resolution to baseline or to a maximum of KDIGO Stage 1 (i.e. Cr of <  = 1.9 baseline); nonrecovery as KDIGO Stage 2/3 or renal replacement therapy.

**Figure 3:**
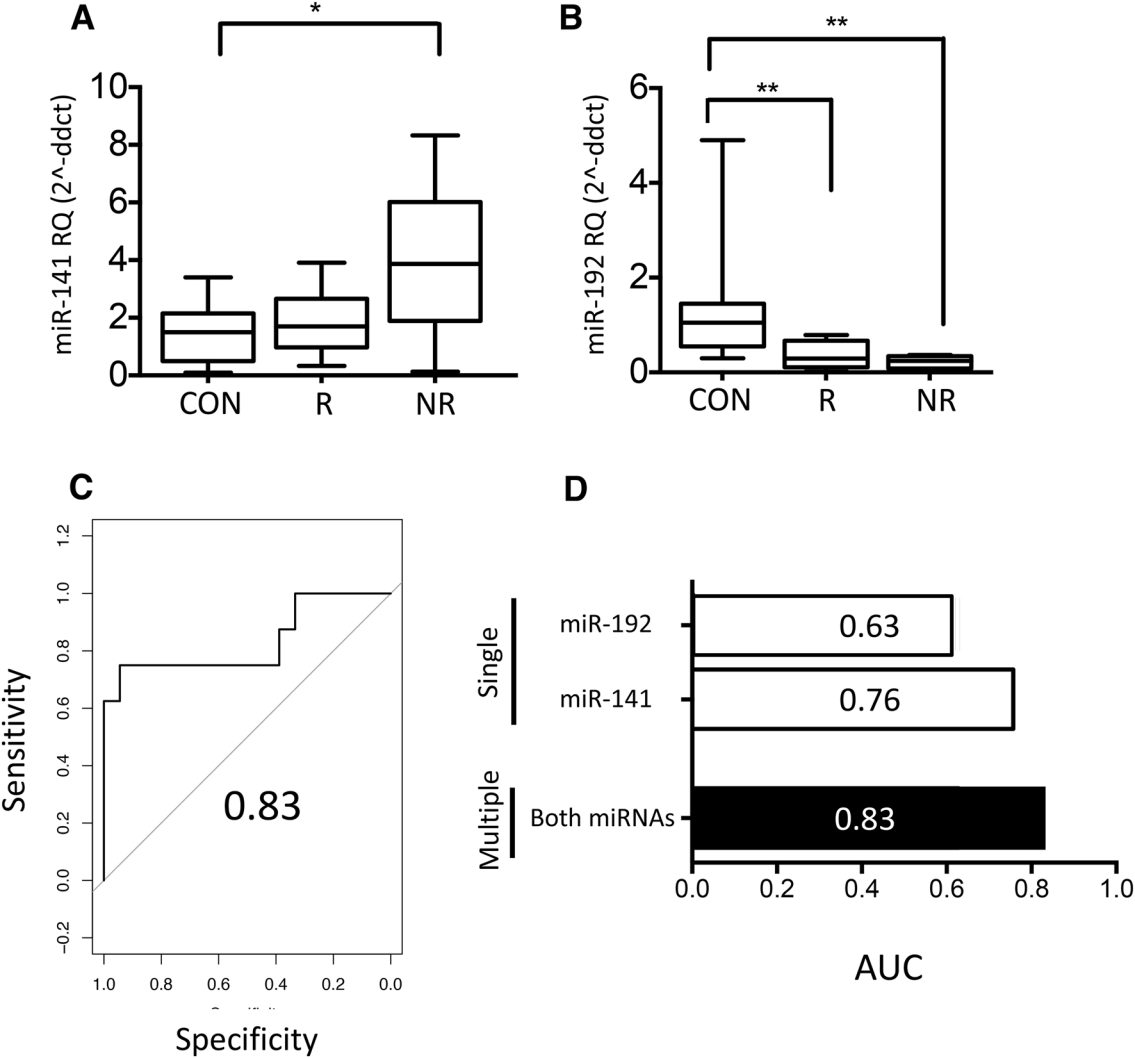
Elevated urinary miR-141 and decreased miR-192 are associated with AKI nonrecovery. (**A**-**D**) At 90 days post study entry, significant differences were observed between recovered (R; n = 18) and nonrecovered (NR; n = 8) AKI patients for (**A**) miR-141 (threefold increase) and (**B**) miR-192 (70% decrease in control compared with nonrecovered). (**C**) Receiver operating characteristic (ROC) curve for miR-141 and miR-192 area under the curve (AUC) = 0.83 in recovered compared with nonrecovered AKI patients. (**D**) Bar graph of AUCs for single and multiple miRNA comparisons between recovered and nonrecovered AKI patients. Statistical analysis comparing two groups was carried out by Mann Whitney U test, for comparison of three or more groups the Kruskal–Wallis test was used with Dunn’s multiple comparisons. Data were normalised to endogenous control miR-191 and are presented as median ± range; *p < 0.05, **p < 0.01, CON: control, R: recovered AKI patients, NR: nonrecovered AKI patients.

Significant differences were observed between controls (n = 10) and nonrecovered AKI patients (n = 8) for urinary miR-141 (threefold increase, p < 0.05) and lowered miR-192 (70% decrease, p < 0.01) (Fig. 3A,B, respectively), but not miR-126, miR-21 and miR-204 (data not shown). RT-qPCR data for miR-141 and miR-192 were used to compare AKI recovery and nonrecovery, with combined receiver operating characteristic (ROC) curve analysis giving an AUC of 0.83 (Fig. 3C). As above, combined analyses performed better than individual miRNA comparisons for miR-141 alone (AUC = 0.63) and miR-192 alone (AUC = 0.76) (Fig. 3D).

### Increased miR-141 and miR-200c in in vitro oxidative AKI models

Previous reports have ascribed functional roles to putative AKI biomarkers in disease^20^. We have shown that miR-192 affects G2/M cell cycle arrest, a key switching point in progression of AKI to chronic kidney disease (CKD)^18,19,21^. In this study, we focused on the role of miR-141 in AKI.

A member of the miR-200 family, miR-141 has an identical seed sequence to miR-200a, also sharing close seed sequence and mature miRNA sequence similarities with other family members miR-200b, miR-200c and miR-429. Frequently, these transcripts function collectively. In the human genome miR-141 and miR-200c are co-transcribed from a chromosome 12 locus; miR-200a, miR-200b and miR-429 from a locus on chromosome 1.

**Figure 4:**
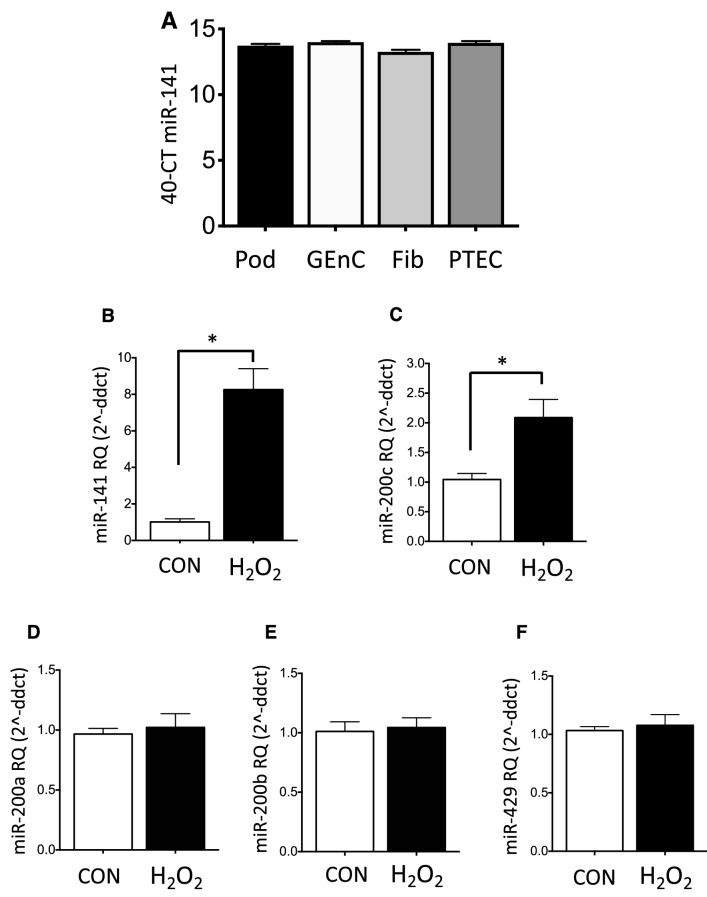
Expression of miR-200 family miRNAs in an in vitro oxidative stress model of AKI. (**A**) RT-qPCR analysis of four cells types found in the kidney: podocytes (Pod), glomerular endothelial cells (GEnC), fibroblasts (Fib) and proximal tubular epithelial cells (PTEC) showed miR-141 detection at similar abundance in each. (**B**-**F**) RT-qPCR data for miR-200 family expression in an in vitro oxidative stress model of AKI, in which PTECs were treated with 1 mM H_2_O_2_ for 24 h. (**B**,**C**) miR-141 and miR-200c were upregulated under oxidative stress (sevenfold and twofold changes, respectively). (**D**-**F**) miR-200a, miR-200b and miR-429 showed no significant changes in this model. Statistical analysis comparing H_2_O_2_-treated PTECs with untreated control cells was carried out by unpaired T-test (n = 3). Data were normalised to endogenous control miR-191 and are presented as mean ± SEM; *p < 0.05.

Using RT-qPCR we analysed miR-141 expression in four renal cell types: podocytes, glomerular endothelial cells, fibroblasts and PTECs. A similar magnitude of detection was observed in each (Fig. 4A). We then analysed PTEC expression of all miR-200 family members using H_2_O_2_-driven cell injury to simulate oxidative stress in vitro. Significant miR-141 upregulation (sevenfold, p < 0.05 Fig. 4B) was seen and miR-200c followed a similar expression pattern with significantly greater abundance in H_2_O_2_-treated PTECs (twofold, p < 0.05, Fig. 4C). MiR-200a, miR-200b and miR-429 showed no significant changes (Fig. 4D-F).

### Forced miR-141 expression in vitro results in increased PTEC death and reduced cell viability

**Figure 5:**
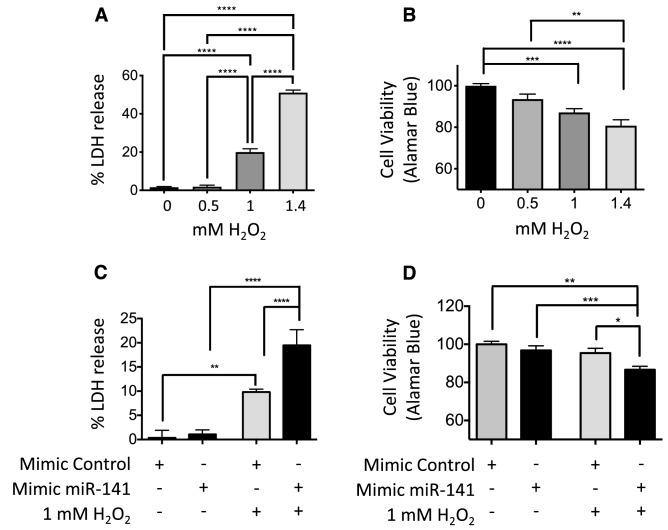
PTEC analysis in vitro: cell death and cell viability under oxidative stress, and manipulation of miR-141 expression. (**A**) Cell death marker lactate dehydrogenase (LDH) increased significantly at 1 mM and 1.4 mM H_2_O_2_. (**B**) Cell viability marker Alamar Blue showed 10% and 20% fewer viable cells in 1 mM and 1.4 mM H_2_O_2,_ respectively. (**C**) A 100% increase in PTEC LDH release was seen in 1 mM H_2_O_2_ with forced miR-141 expression, with no significant change following forced miR-141 expression alone. (**D**) Forced miR-141 expression resulted in a 10% decrease in PTEC viability compared with miRNA mimic control under 1 mM H_2_O_2_, with no significant change following forced miR-141 expression alone. Statistical analysis between groups was carried out using one-way ANOVA with post-hoc analysis of Turkey. Data are presented as mean ± SEM; *p < 0.05, **p < 0.01, ***p < 0.001, ****p < 0.0001.

PTEC death is a key pathological marker post-AKI, and our in vitro oxidative stress model mimicked this phenotype. We observed a dose-dependent increase in release of lactate dehydrogenase (LDH), used here as a marker of cell death, and Alamar Blue analysis showed decreased cell viability in response to increased H_2_O_2_ (Fig. 5A,B).

In this study we have shown that PTEC miR-141 expression increases significantly in response to oxidative stress (Fig. 4B). To model the functional effects of this increase in AKI, forced miR-141 expression in PTECs in the presence of 1 mM H_2_O_2_ was compared to addition of control miRNA mimics (Fig. 5C,D). No significant effects were seen in the absence of H_2_O_2_, but in 1 mM H_2_O_2_, a significant increase in cell death (100%, p < 0.001, Fig. 5C) and decrease in cell viability (10%, p < 0.05, Fig. 5D) were observed.

### Identification of miR-141 targets that increase cell death and reduce cell viability under oxidative stress

MiR-141 inhibits expression of ZEB1 and ZEB2, leading to derepression of E-cadherin expression (e.g. Supplementary Fig. S1a,b online), and repression of miR-141 upregulates ZEB expression (e.g. Supplementary Fig. S1c,d online), which may lead to epithelial-mesenchymal transition via a ZEB1/2-dependent pathway. Here we analysed PTECs following H_2_O_2_-treatment and showed a significant increase in ZEB2 mRNA (twofold increase, p < 0.01, Supplementary Fig. S1e online) and significant decrease of E-cadherin transcription (60% decrease, p < 0.001, Supplementary Fig. S1f. online). These data suggest that the injury effects seen with increased miR-141 expression in H_2_O_2_ are not ZEB-mediated.

MiRNAs may have several hundred potential mRNA targets^22–24^. To identify potential miR-141 target mRNAs in our oxidative stress model, we first carried out in silico analysis using four target prediction algorithms: DIANA (http://snf-515788.vm.okeanos.grnet.gr/)22, miRDB (http://mirdb.org/)23, miRanda (http://www.microrna.org/microrna/home.do)24, and Target Scan (http://www.targetscan.org/vert_71/)25.

**Figure 6:**
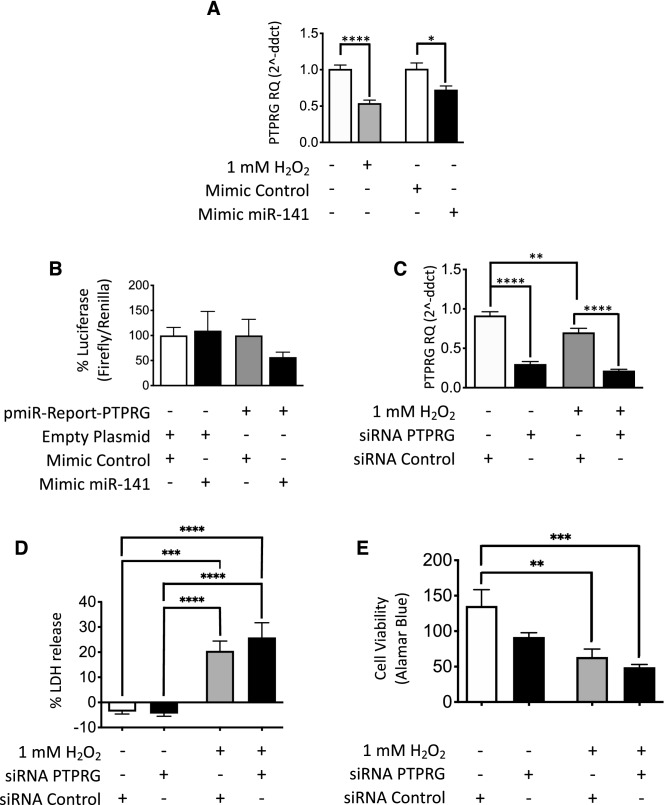
Luciferase reporter analysis of miR-141: PTPRG 3′-UTR interaction and siRNA knockdown of PTPRG in PTECs in response to oxidative stress and forced miR-141 expression. (**A**) RT-qPCR analysis of PTPRG showed significant downregulation in 1 mM H_2_O_2_ (50% decrease) and following forced miR-141 expression (30% decrease). (**B**) PTPRG 3′-UTR reporter assay activity in PTECs showed PTPRG to be a direct miR-141 target. (**C**) RT-qPCR analysis showed significant PTPRG mRNA knockdown using siRNA, as well as repression of PTPRG in 1 mM H_2_O_2_ compared to healthy cells. (**D**) LDH release increased significantly under oxidative stress and showed a further increase with PTPRG knockdown. (**E**) Cell viability marker Alamar Blue decreased in 1 mM H_2_O_2_, lowering further following PTPRG knockdown. Statistical analysis between groups was carried out using one-way ANOVA with post-hoc analysis of Tukey (n = 3). Data are presented as mean ± SEM; *p < 0.05, **p < 0.01, ***p < 0.001, ****p < 0.0001.

Data from each algorithm were compared to identify miR-141 targets predicted by all four (Supplementary Fig. S2 online). These findings were then analysed to find miR-141-specific mRNA targets with 2 or more miRNA seed sequence binding sites. Nine mRNAs were identified, seven of which were detected in PTECs by RT-qPCR. Of these seven, DEK proto-oncogene (DEK), transcription factor 12 (TCF12), and LIM domain only 3 (LMO3) showed no change following forced miR-141 expression or 1 mM H_2_O_2_ stimulation (Supplementary Fig. S3 a,b,c online). RT-qPCR mRNA detection for protein kinase cAMP-activated catalytic subunit β (PKRACB), transcriptional adaptor 1 (TADA1), protein phosphatase 1F (PM1EF) decreased following forced miR-141 expression (Supplementary Fig. S3d,e,f, online). As shown in Fig. 6A, only protein tyrosine phosphatase receptor type G (PTPRG) mRNA showed the same significant decrease in abundance in the presence of 1 mM H_2_O_2_ (50% decrease, p < 0.0001) and following forced miR-141 expression (30% decrease, p < 0.05).

### Analysis of renal PTPRG expression

PTPRG is a member of the protein tyrosine phosphatase family. Using RT-qPCR we detected PTPRG mRNA in podocytes, glomerular endothelial cells, fibroblasts and PTECs (Supplementary Fig. S4a online). Global human and mouse RNA libraries were then analysed (Supplementary Fig. S4b,c online, respectively) and PTPRG mRNA synthesis was normalised to GAPDH and expressed as fold change relative to the organ with the lowest PTPRG detection. Human renal PTPRG expression was 15-fold greater than in skeletal muscle, in mouse kidneys a 100-fold more was seen in comparison to peritoneal expression (Supplementary Fig. S4 b,c online).

### PTPRG is a direct target of miR-141; siRNA PTPRG knockdown results in increased PTEC death and decreased cell viability in vitro

Luciferase reporter construct pmiR-Report-PTPRG, containing the full length PTPRG 3′-untranslated region (3’-UTR), was used to analyse miR-141 interactions. As shown in Fig. 6B, cotransfection of pmiR-Report-PTPRG and mimic miR-141 led to a reduction in luciferase activity compared to cotransfection of pmiR-Report-PTPRG and control mimic.

To investigate the functional effects of manipulating PTPRG expression in PTECs, siRNA knockdown was used. RT-qPCR analysis confirmed a 70% decrease in mRNA detection, and the same direction of effect was observed under oxidative stress (Fig. 6C). As shown in Fig. 6D, LDH release increased significantly in the presence of H_2_O_2_ and a further increase was observed following PTPRG knockdown. Figure 6E shows decreased cell viability in 1 mM H_2_O_2_ and a further decrease following PTPRG mRNA knockdown.

### Validation of miR-141 upregulation and PTPRG suppression in vivo

**Figure 7:**
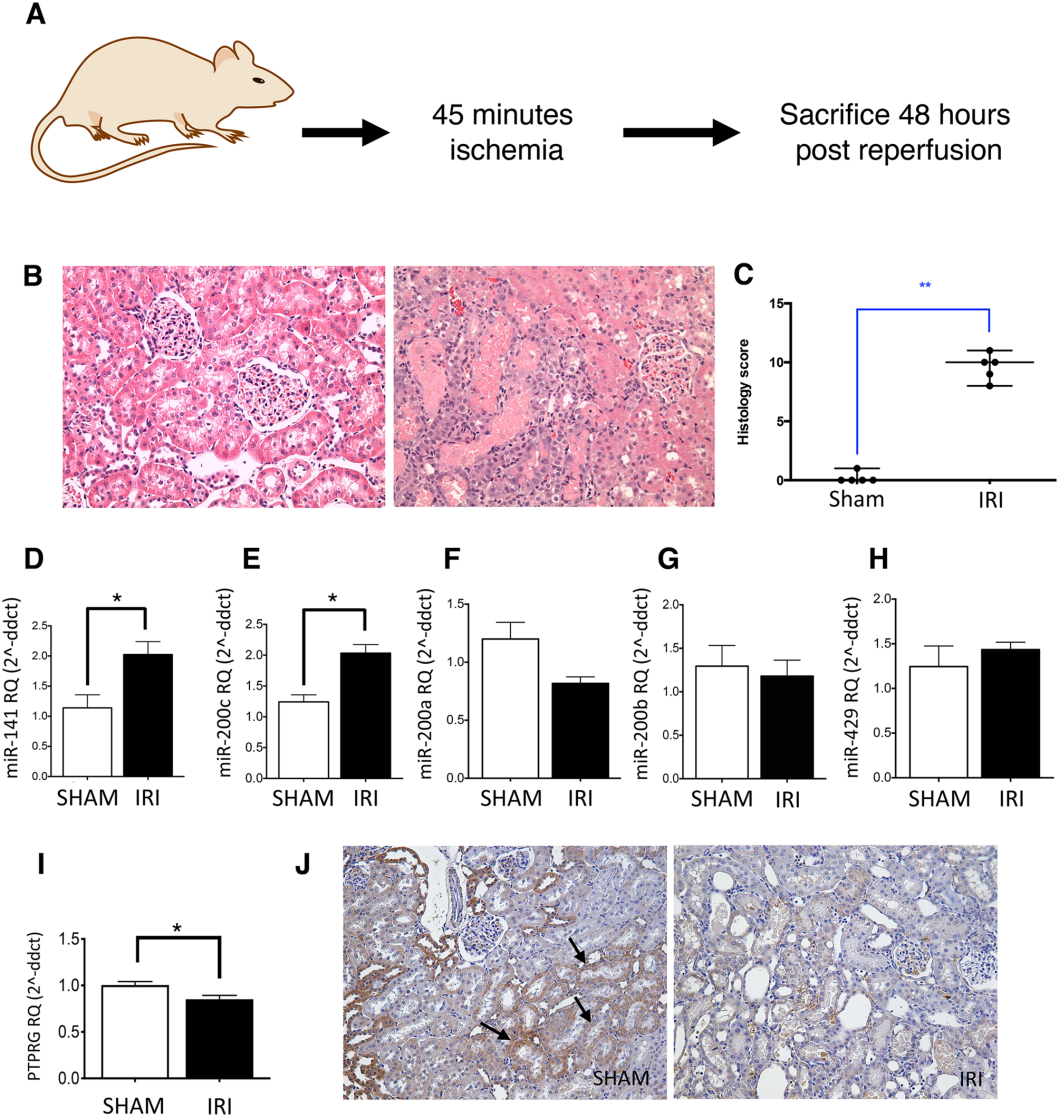
MiR-200 family expression in a rat unilateral ischaemic reperfusion injury (IRI) model of AKI. (**A**) A diagram depicting our unilateral IRI model in Lewis male rats and (**B**,**C**) histological evidence of tubular damage. (**D**-**H**) RT-qPCR analysis of the miR-200 family expression in rat kidneys from animals in sham and IRI experimental groups. Detection of miR-141 and miR-200c was upregulated in the unilateral IRI model (twofold, **D**,**E**), while miR-200a, miR-200b and miR-429 showed no significant changes between sham and unilateral IRI groups (**F**–**H**). RT-qPCR analysis of PTPRG expression in whole kidney showed a significant injury-specific decrease in PTPRG expression (**I**). Histological analysis demonstrated clear kidney tubular PTPRG expression in sham tissue sections, including expression in PTECs (e.g. see arrows), which was not present in IRI tissue sections (**J**). Statistical analysis for IRI compared to controls was carried out by unpaired T-test (n = 4). Data were normalised to endogenous control miR-191 and are presented as mean ± SEM; *p < 0.05, **p < 0.01.

To investigate our findings in vivo, expression of miR-200 family members was analysed in kidney samples from our unilateral ischemic reperfusion injury (IRI) rat model of AKI (Fig. 7A-C)^26^. RT-qPCR data shown in Fig. 7D,E showed the same increase in miR-141 and miR-200c that we observed in our in vitro model (p < 0.05), and similarly that no changes were observed in the other miR-200 family members (Fig. 7F-H). A significant reduction in renal PTPRG mRNA expression was observed following unilateral IRI compared to control animals (Fig. 7I, p < 0.05). Immunohistochemical analysis revealed PTPRG expression in kidney tubules (Fig. 7J).

## Discussion

Current biomarkers are unable to show real-time changes in renal function during AKI, and have limited ability to classify disease and stratify rapidly progressing patients. In this study we hypothesised that altered urinary miRNA profiles could predict AKI recovery/nonrecovery after 90 days, and that AKI-specific PTEC changes would identify miRNAs that mediated AKI pathology. To test our hypothesis, we used an unbiased miRNA profiling approach to identify urinary miRNAs that would predict 90 day recovery/nonrecovery, and then manipulated expression of selected miRNAs in injury models to investigate underlying AKI mechanisms.

Profiling analysis in our screening cohort identified fourteen miRNAs with altered abundance in recovery/nonrecovery. We confirmed increased detection of urinary miR-21, miR-126 and miR-141, and decreased detection of urinary miR-192 and miR-204 in our complete AKI patient cohort compared with healthy controls. Further analysis highlighted significantly increased detection of urinary miR-141 and significantly decreased detection of miR-192 in nonrecovered AKI patients.

Sonoda and colleagues (2019) reported that extracellular-vesicle-associated urinary miRNA quantification might provide data to aid patient stratification^27^. These workers profiled urinary exosome miRNAs from a rodent bilateral IRI model of AKI at multiple timepoints and divided their findings into injury, early recovery and late recovery phases. Increased abundances of urinary miR-141 and other miR-200 family members were observed in early recovery^27^. However, while this study highlighted the potential utility of these miRNAs as biomarkers of AKI nonrecovery, the data require validation in patient samples.

In addition to their predictive value, biomarkers can also provide valuable insights into disease mechanisms, thereby identifying novel targets with therapeutic potential. AKI biomarkers are frequently divided into three broad categories: (i) constitutively expressed proximal tubular proteins, (ii) tubular stress molecules which respond to injury with increased gene expression and (iii) low molecular mass plasma proteins that are filtered freely at the glomerulus^28^.

Work from this laboratory has shown decreased miR-192 detection in biopsy samples from diabetic nephropathy patients was associated with increased renal fibrosis and decreased renal function as estimated glomerular filtration rate (eGFR)^29^. We have also observed that forced miR-192 expression in PTECs opposed TGF-β1-mediated fibrotic mechanisms by preventing critical changes in cell phenotype^30^. In an in vitro aristolochic acid model of AKI in PTECs, we showed that miR-192 induced PTEC growth arrest in the G2/M phase of the cell cycle^18^.

Cell cycle is key to AKI progression, early stage damage results in G1 phase arrest, more severe damage causes arrest in G2^31^. There is increasing appreciation that AKI followed by incomplete repair represents a major risk factor for subsequent renal fibrosis and progression to CKD. Yang and co-workers highlighted a link between G2/M cell cycle arrest and fibrosis in PTECs^19^, and demonstrated that G2/M cell cycle arrest affects downstream fibrotic signaling in a p53-dependent manner as we have also shown^18,19^.

In the next stage of our study we focused on the role of miR-141 in AKI progression. MiR-141 is a member of the miR-200 family and is co-transcribed with miR-200c. Numerous studies have described a protective role for miR-200 family members, maintaining epithelial cell phenotype by downregulating expression of transcription factors ZEB1 and ZEB2, thereby derepressing E-cadherin expression^32^.

MiRNAs repress gene expression by interaction with specific sequences in the 3′-UTRs of target mRNAs. The key miRNA sequence motif involved in the recognition process, known as the seed sequence, differs by only one nucleotide between miR-141 and miR-200c. It is therefore conceivable that these miRNAs may share downstream mRNA targets.

In this study we showed that miR-141 was detected at equal abundance in podocytes, glomerular endothelial cells, fibroblasts and PTECs. The renal proximal tubule is the principal site of injury following AKI, and tubular recovery is key to restoring kidney function^33^. We observed upregulated miR-141 and miR-200c expression in PTECs under H_2_O_2_-mediated oxidative stress with no effect on other miR-200 family members, and subsequently replicated this finding in our rat unilateral IRI model. Since overall kidney function is not affected in our unilateral IRI model, further analyses will be required to investigate the relationship between miR-141/miR-200c expression and kidney function in injury and repair/recovery.

H_2_O_2_ caused a dose-dependent increase in cell death that increased further with forced miR-141 expression. Oxidative stress also decreased cell viability, and forced miR-141 expression led to a further decrease. We identified mRNAs with miR-141 recognition sequence motifs in silico. Subsequent in vitro analysis of seven mRNAs that were expressed in PTECs identified PTPRG as a miR-141 target.

PTPRG is a member of the protein tyrosine phosphatase family, one of two major groups of enzymes that regulate tyrosine phosphorylation/dephosphorylation. PTPRG dephosphorylates tyrosine residues, switching off downstream signalling. An important tumor suppressor gene, PTPRG is frequently deleted in breast, nasopharyngeal carcinoma and renal cell cancers^34–36^. Overexpression of PTPRG in cancer cells inhibits cell proliferation, while its repression increases proliferation. However, our data showed that forced miR-141 expression and subsequent PTPRG suppression resulted in increased PTEC death and decreased cell viability.

In AKI, death of badly damaged PTECs is followed by proliferation and migration of remaining healthy cells, restoring tubular integrity and kidney function. We therefore hypothesise that suppression of PTPRG expression could affect cellular proliferation at a later/subsequent time point than those analysed in our experiments, and that this avenue warrants further investigation.

Overexpression of PTPRG in cancer also delays cell cycle re-entry due to increased expression of cell cycle regulators p21^cip^ and p27^kip^ ^36^. While this process may protect cells entering cell division with damaged DNA^31^, prolonged cell cycle arrest leads to senescence and subsequent fibrosis, potentially facilitating AKI progression to CKD^31^.

PTPRG is expressed in many different organs. Zhou et al. (2016) determined that PTPRG is expressed at the PTEC basolateral membrane and plays an important role in maintaining extracellular CO_2_/HCO_3_ sensing mechanisms critical for pH homeostasis^37^.

PTPRG interacts with epidermal growth factor receptor (EGFR), a protein tyrosine kinase containing several phosphorylation sites, and dysregulated EGFR expression has been linked to renal disease. Dephosphorylation of EGFR receptor at Y-1068 and Y-1086 in nasopharyngeal carcinoma inactivates the PI3K/Akt signaling cascade, resulting in the downregulation of proangiogenic pathways, invasive proteins including VEGF, IL-6 and IL-8, and suppressing tumour cell proliferation^34^. Shu et al. showed that PTPRG overexpression affects cell cycle in breast cancer via ERK1/2, a downstream effector of EGFR signalling^36^.

**Figure 8:**
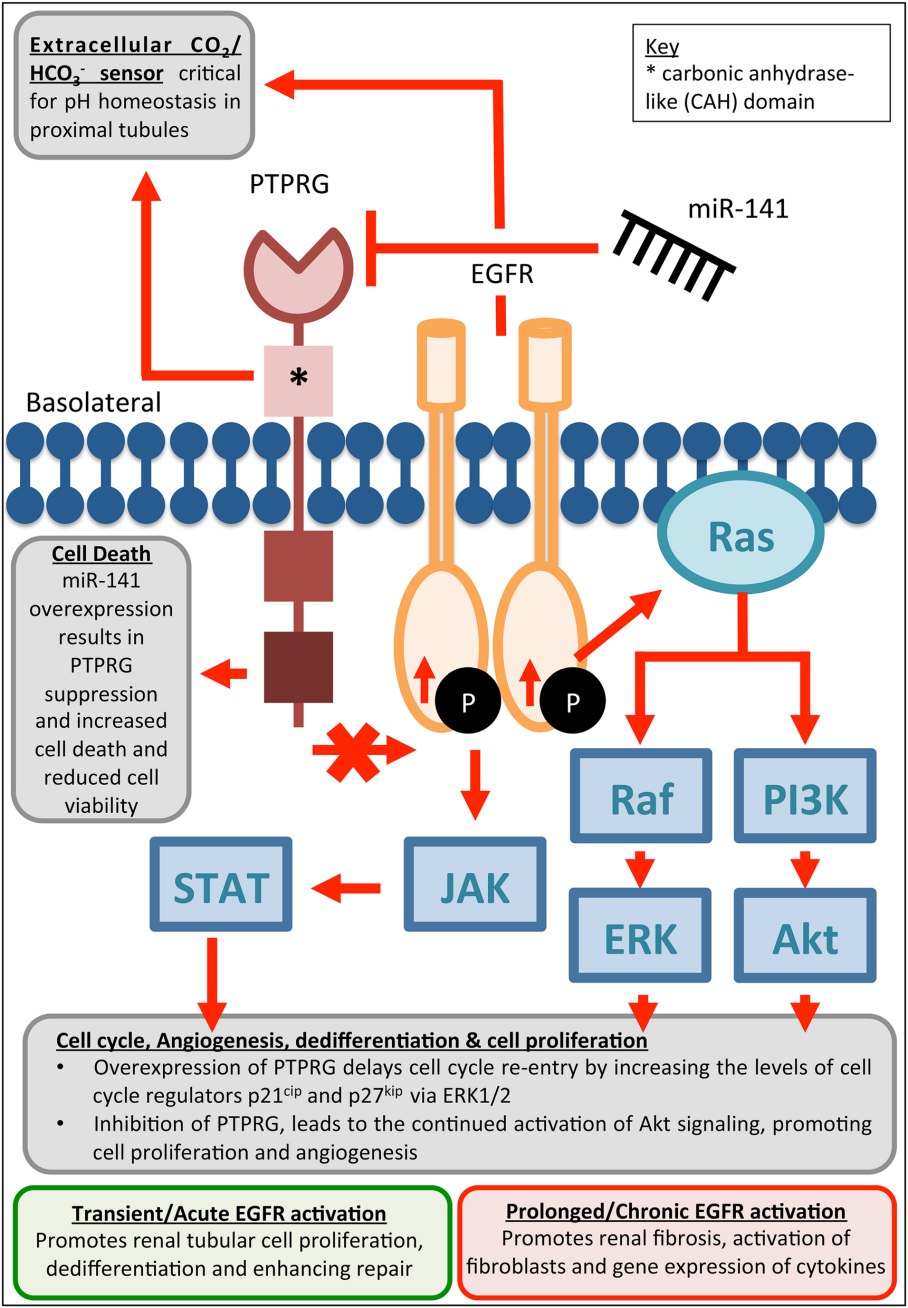
Hypothetical miR-141 regulation of PTPRG expression and its effect on PTEC signalling.

EGFR responses in the kidney depend on the precise tissue and cell environment^36^. Animal models of AKI have shown that EGFR activation promotes renal tubular cell proliferation, and enhances recovery of renal function and structure following AKI^38,39^. Zhuang and colleagues demonstrated increased EGFR phosphorylation in H_2_O_2_-treated PTECs^40^. However, prolonged EGFR activation in the kidney can be profibrotic, resulting in the renal interstitial fibroblast activation and expression of multiple profibrogenic cytokines including TGF-β^38^. It is therefore possible that transient PTPRG suppression by miR-141 could result in EGFR activation and accelerate renal recovery, but that prolonging this effect results in chronic EGFR activation and progression of a fibrotic phenotype, as summarised in Fig. 8.

In summary, we used unbiased profiling to determine that miR-21, miR-126, miR-141, miR-192 and miR-204 showed promise as AKI biomarkers, and that miR-141 and miR-192 were associated with AKI nonrecovery. Forced miR-141 expression repressed PTPRG expression and increased PTEC death in models of acute injury, which has been the focus of this study. Further work will be required to determine the relationship between PTPRG and EGFR in the kidney, and to investigate the impact of transient and prolonged PTPRG suppression in renal injury, repair and recovery.

## Methods

### Study participants

AKI was defined by an increase in serum creatinine (SCr) of ≥ 26.5 µmol/l within 48 h; or an increase in SCr to ≥ 1.5 × baseline, which is known or presumed to have occurred within the prior 7 days; or urine volume < 0.5 ml/kg/h for 6 h. Further to this, AKI is staged for severity into three groups: Stage 1, 2 and 3. This study included stage 3 AKI patients defined as 3.0 × baseline; or with an increase in SCr to ≥ 353.6 µmol/l; or initiation of renal replacement therapy.

Our initial profiling study cohort of 6 recovered and 5 non-recovered AKI patients was obtained from the Wales Kidney Research Tissue Bank, University Hospital of Wales (Cardiff, UK). The subsets of patient samples selected for pooling represented those most adherent to our definitions of recovery and nonrecovery. As we have described previously, archived control data (n = 20) Gene Expression Omnibus (https://www.ncbi.nlm.nih.gov/geo; accession number GSE114477)^12^ was used for comparison between AKI patients and controls in the profiling experiment. The definition of recovery at 90 days we used was resolution to baseline or to a maximum of KDIGO Stage 1 (i.e. Cr of <  = 1.9 baseline); nonrecovery was defined as KDIGO Stage 2/3 or renal replacement therapy.

As seen in Table 1, our replication cohort included the 11 AKI patients from the profiling cohort along with a further 18 AKI patients, to give a total AKI cohort of n = 29. The AKI group was 56.7% male, their mean age was 62.4 with a mean creatinine at entry to the study of 493.6 mg/mmol (SD, ± 283.4 mg/mmol). The control group of 10 unaffected individuals was obtained from the Wales Kidney Research Tissue Bank, University Hospital of Wales, Cardiff, UK. Patient demographics and clinical parameters are shown in Table 1. All patients were recruited from renal inpatients at the University Hospital Wales, Cardiff, UK, and all methods were carried out in accordance with relevant guidelines and regulations. Informed consent was obtained by the Wales Kidney Research Tissue Bank for both patient and control samples. The Wales Kidney Research Tissue Bank Governance Committee granted ethical approval for this study.

### Urine collection, urinary RNA isolation, and RT-qPCR analysis

Urine samples were taken from patients who presented with stage 3 (advanced) AKI on admission to our hospital, were sampled at presentation, and outcome was measured at 90 days. Urine samples were collected, and RNA extraction from 350 μL of urine, generation of cDNA from equal volumes of RNA extracts, and reverse transcription-quantitative PCR (RT-qPCR) were then performed essentially as we have described previously^11,12,41^. Each miRNA was detected by specific Taqman assay (Thermo Fisher Scientific, Paisley, UK): hsa-miR-21-5p (identification number 000397), hsa-miR-126-3p (000413), hsa-miR-141-3p (000463), hsa-miR-191-5p (002299), hsa-miR-192-5p (000491) and hsa-miR-204-5p (000508). Relative quantification was calculated using the 2^-ΔΔCt^ method, with miRNA expression normalised to hsa-miR-191-5p^12^.

### MiRNA profiling by Taqman Low Density Array (TLDA) human miRNA cards

Urinary miRNAs were reverse transcribed using the Megaplex Primer Pool (Human Pools A version 2.1; Thermo Fisher Scientific) with a predefined pool of 381 reverse transcription primers. A fixed volume of 3 μL of RNA solution was used as input in each RT reaction, and RT performed according to the manufacturer’s recommendations. Reverse transcription reaction products were amplified using Megaplex PreAmp Primers (Primers A version 2.1; Thermo Fisher Scientific), the samples were then diluted to a final volume of 100 μL, and pools of 6 AKI recovered patients and 5 AKI nonrecovered patients analysed on one TLDA A card. Findings were compared to archived control values described elsewhere^12^*.*

The TLDA Card A version 2.1 (Thermo Fisher Scientific) was used to quantify 377 human test miRNAs, three endogenous controls and a negative control. Quantitative PCR was performed on an Applied Biosystems 7900HT thermocycler (ThermoFisher Scientific) using the manufacturer’s recommended program. MiRNA profiling data were analysed using global normalisation and analysed for > twofold differences between groups.

### Cell culture and H_2_O_2_ treatment

We have described culture of human fibroblast and conditionally immortalised human glomerular endothelial cell and podocyte cell lines elsewhere^11^. Human renal proximal tubular epithelial cells (PTECs) from cell line HK-2 were cultured in Dulbecco’s modified Eagle’s medium/Ham’s F12 medium supplemented with 10% foetal calf serum (FCS), 20 mmol/L HEPES, 5 μg/ml transferrin, 40 ng/mL hydrocortisone and 5 μg/ml sodium selenite (BioIVT, Burgess Hill, West Sussex, UK). Cells were grown at 37 °C in 5% CO_2_ and 95% air. Growth medium was replenished every 3 to 4 days until cells were confluent. Cells were then plated into 12, 48 or 96 well plates for experimentation, and serum starved overnight at 80–90% confluence prior to treatment with 0.5–1.4 mM H_2_O_2_.

### PTEC miRNA transfection

For miRNA gain- and loss-of-function experiments, HK-2 cells at 60% confluence were transfected with miRVana miRNA mimics (5 nmol/L) or inhibitors (50 nmol/L; Thermo Fisher Scientific) prior to serum starvation or H_2_O_2_ treatment. Lipofectamine RNAiMAX transfection reagent (Thermo Fisher Scientific) was used according to the manufacturer’s protocol. For experiments using siRNAs (20 nmol/L; Thermo Fisher Scientific) HK-2 cells were transfected using Lipofectamine RNAiMAX at 60–80% confluence with PTPRG-specific Silencer Select siRNA (cat: 4,392,420, assay ID: s11549) or control (cat: 4,390,843).

### Unilateral ischaemic reperfusion injury (IRI) model

The IRI model was performed as we have described elsewhere^26^. In brief, rats (n = 5 per group) were anesthetised with isoflurane, a midline laparotomy incision made, and the left renal pedicle was identified and clamped for 45 min using a vascular clip (IRI group). The kidney was visually assessed for both ischaemia upon clamping and reperfusion upon release of the clamp. Sham group animals underwent the same procedure without clamping. Kidneys were retrieved 48 h after terminal anaesthesia. All animal experiments were conducted according to the United Kingdom Use of Animals (Scientific Procedures) Act 1986, under licence PPL30/3097. The rats were given a 7-day period of acclimatisation to their new surroundings, and were housed and handled according to the local institutional policies and procedures licenced by the Home Office. Ethical approval for all the protocols within the Licence was provided by the Animal Welfare and Ethical Review Body under the Establishment Licence held by Cardiff University. All experiments involving the use of animals were carried out in accordance with the ARRIVE guidelines.

### RNA extraction, RT and qPCR from animal tissues and cells

Animal tissues were homogenised in TRI Reagent (Merck, Darmstadt, Germany) using a hand-held homogeniser, while cells were lysed by addition of TRI Reagent directly to the culture plate, and the surface scratched with a 1 mL pipette tip. Homogenised/lysed samples were collected in 1.5 mL tubes and stored at -80 °C for later extraction. Prior to RNA extraction, samples were thawed on ice then left at room temperature for 5 min. Chloroform was added to each sample, tubes were inverted to mix their contents and the samples were centrifuged at 12,000 g for 15 min at 4 °C. Each aqueous phase was then transferred to a fresh 1.5 mL tube, the RNA precipitated using isopropanol and the pellet washed with 75% ethanol three times prior to air drying and dissolution in water. RNA concentrations were quantified and 10 ng was added to each miRNA reverse transcription (RT) reaction, 250–1000 ng was added to mRNA RT reactions. RT reactions were carried out using the High Capacity cDNA Kit (Thermo Fisher Scientific) as recommended by the manufacturer, and miRNAs analysed using Taqman assays (Thermo Fisher Scientific) as described above. For mRNAs, primers (see Table 2) were designed using Primer-BLAST and qPCR performed using power SYBR green according to the manufacturer’s protocol. Data were analysed using the 2^–∆∆Ct^ method^12^.

**Table 2:** Oligonucleotide primer sequences for SYBR Green qPCR.

Primer name	Forward primer sequence	Reverse primer sequence
GAPDH	CCTCTGACTTCAACAGCGACAC	TGTCATACCAGGAAATGAGCTTGA
ZEB1	CAGGCAGATGAAGCAGGATGT	TGACAGCAGTCTTGTTGTTGTAG
ZEB2	CCTTCTGCGACATAAATACGAACA	AGTACGAGCCCGAGTGTGAGA
ECADHERIN	TCCCAATACATCTCCCTTCACA	ACCCACCTCTAAGGCCATCTTT
PRKCAB	CTGCCTTATGGGACAGATCAA	GTCCGGCATTATTCTGAGTTGG
TADAL1	CCGCAGCCATACCTGAAGAA	CAGTAAAAGCTGGAGGGCTTTC
DEK	TCTTCCTTACAGAGAGAGCCATT	GTTTCTGCCCCTTTCCTTGTG
TCF12	CTAGAGGCAGAACAAGCAGT	CTGGCATTGTTAGCCATCCG
LMO3	ACTATCTGAGGCTCTTTGGTGT	AAACATTGTCCTTGGACGC
PM1EF	AAGCAGCCAGGGAGAGCTTA	AGTTCAACAGCTTGGCCCTT
Hsa-PTPRG	CAGGACCATAGGCACCAGAG	CCGTGCTCCATTATGTCGTG
Rn-PTPRG	ACCAGTATTGGCCAACGAG	GGTGTAGCAGGCGTGTACTT
mm-PTPRG	TCAGTCAATGCCGGGAAGCA	TCGCTCGGAAGCAAGCAAGAACTTAT

### PTPRG 3’-UTR amplification

The PTPRG 3′-UTR, spanning 4.306 kb, was PCR-amplified as three fragments, P1-P3 (see Table 3. For primers sequences). PCR primers were designed according to the manufacturer’s recommendations for use with the In-Fusion HD Cloning Kit (Takara Bio, Saint-Germain-en-Laye, France) and reactions were carried out using CloneAmp HiFi PCR Premix (Takara Bio, Cat. No. 639298). For fragments P1 and P2, 4 µL of diluted cDNA (10 ng of RT-RNA) from control HK-2 cells was mixed with 0.25 µM of each primer, 12.5 µL of CloneAmp HiFi PCR premix in a 25 µL reaction volume. An initial denaturing step of 2 min at 98 °C was followed by forty cycles of PCR amplification consisting of 15 s at 98 °C, 30 s at 56 °C and 2 min at 72 °C, and then a final 5 min at 72 °C before cooling to 4 °C. Fragment P3 was amplified from human genomic DNA using the same PCR conditions described above. All PCR amplification products were visualised and sized by electrophoresis in 1% (w/v) agarose, with those of predicted size then excised in a minimal quantity of agarose and column purified using the QIAquick gel extraction kit (Cat. No. 28706, Qiagen, Manchester, UK).

**Table 3:** Oligonucleotide primer sequences for PCR amplification of the PTPRG 3′-UTR.

Primer ID	Primer sequence (5’-3’)	Insert number
P1 _F	CCTATGATATCACGCACGCGCTGGAATCCTGAAAGGGCACT	Insert P1
P1 _R	AACCTCAGTGCTGATCATCATTAATGTTTAATGCA	
P2 _F	TGATGATCAGCACTGAGGTTCTATTTATCTTGATT	Insert P2
P2 _R	TAACCCTTCTTTGGCCTCGCTGGACTCT	
P3_F	GCGAGGCCAAAGAAGGGTTACGGTGTTCAA	Insert P3
P3_R	CATAGGCCGGCATAGACGCGTGAATACATCAAAAACCATACTTT	

### Reporter plasmid digestion and ligation

One µg of p-miR-Report vector was digested with 1,000 U of *Mlu*l (1 µL) in a 50 µL reaction, containing New England Biolabs (Hitchin, Hertfordshire, UK) 3.1 buffer and water. The reaction was incubated at 37 °C for 15 min and then inactivated at 80 °C for 20 min. The linearised p-miR-Report and three inserts (P1, P2, P3) were ligated in a 1:1:1:1 ration using In-Fusion enzyme according to manufacturer’s instructions. Samples were incubated at 50 °C for 15 min and then stored at -20 °C. Following transformation of competent *E. coli* by standard means, individual colonies were picked, digested appropriately to confirm insertion and sequenced.

### PTEC transfection with luciferase reporter vector containing the PTPRG 3′-UTR

Using Lipofectamine LTX (Thermo Fisher Scientific), PTECs at 60% confluence were transfected with p-miR-Report-PTPRG or empty plasmid, and transfection control *Renilla* reporter vector at a ratio of 10:1. The next day cells were transfected with miRVana mimic or control as described above. Following overnight serum starvation, cells were treated with 1 mM H_2_O_2_ for 24 h. Firefly and *Renilla* luciferase activities were quantified using the Dual Luciferase Reporter kit according to the manufacturer's recommendations (Promega, Southampton, UK).

### Immunohistochemistry (IHC) staining

Formalin-fixed paraffin-embedded kidney sections were used for IHC staining. Sections were dewaxed and rehydrated in xylene and ethanol, then blocked with 3% hydrogen peroxide and Ultra V block. Staining with primary PTPRG antibody (ThermoFisher Scientific, PA5-67,565) was followed by primary antibody enhancer, HRP polymer (Thermo Scientific, 12,624,007), DAB and hematoxylin counter stain.

### Statistical analysis

Statistical analyses were performed using GraphPad Prism Version 7 software (La Jolla, CA, USA). Data were expressed as either median (and range) or mean (± SEM) for non-normally distributed and normally distributed data, respectively. Differences between two individual experimental groups of non-normally distributed values were compared using the Mann–Whitney U test, or for experiments with 3 or more groups, the Kruskal–Wallis test followed by post hoc Dunn’s test for individual comparisons. Differences between two individual experimental groups of normally distributed values were compared by two-tailed t-test, or for experiments with 3 or more groups, analysis of variance, two-tailed t-test followed by post hoc Turkey test for individual comparisons. Differences for which p < 0.05 were considered statistically significant. Post hoc analysis using G*Power 3.1 software calculated that a power of 0.8 was achieved to detect a statistical significance level of 0.05 for miR-141 and miR-192^42^.

## Supplementary Information

Supplementary Information.
