# Supplementary material for: miR-141 mediates recovery from acute kidney injury
Source: Sci Rep. 2021 Aug 13;11:16499. doi: 10.1038/s41598-021-94984-x (PMC8363617; doi:10.1038/s41598-021-94984-x)
Supplement: Supplementary file 1 — Supplementary Information. [file 41598_2021_94984_MOESM1_ESM.pdf]

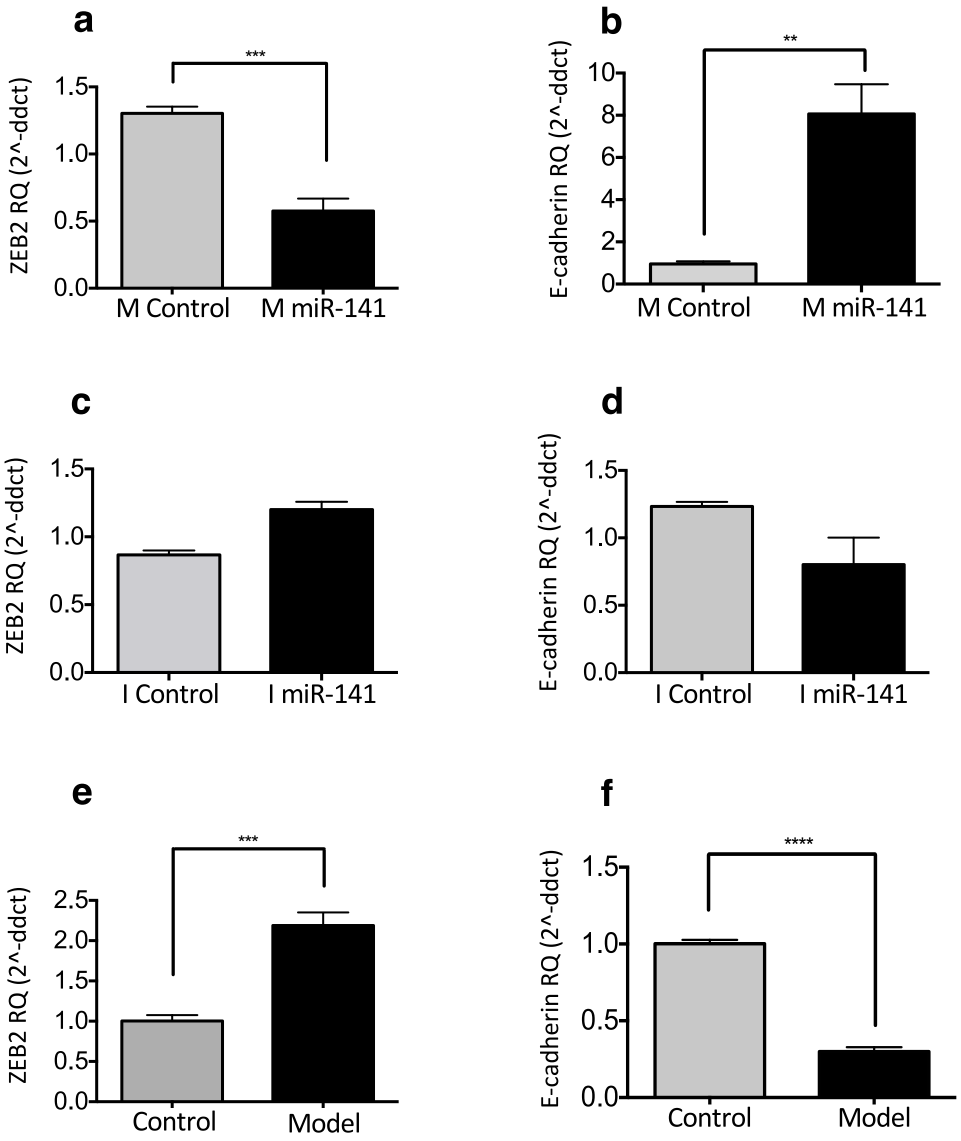

Supplementary Figure 1.

ZEB2 and E-cadherin expression in PTECs following manipulation of miR-141 expression.

(**a**) RT-qPCR analysis showed that forced expression (M) of miR-141 compared to control repressed ZEB2 (60% decrease) and (**b**) led to derepression of E-cadherin (6-fold increase). (**c**) Inhibition (I) of miR-141 upregulated ZEB2 expression (0.3-fold increase) resulting in (**d**) a 20% decrease in E-cadherin expression. (**e**) PTEC treatment with 1 mM H_2O_2 increased ZEB2 expression (1-fold) and (**f**) repressed E-cadherin expression (60% decrease). Statistical analysis of treated compared to controls was carried out by unpaired T-test (n = 3). Data were normalised to endogenous control GAPDH and are presented as mean +/- SEM; **p<0.01, ***p<0.001, ****p<0.0001.

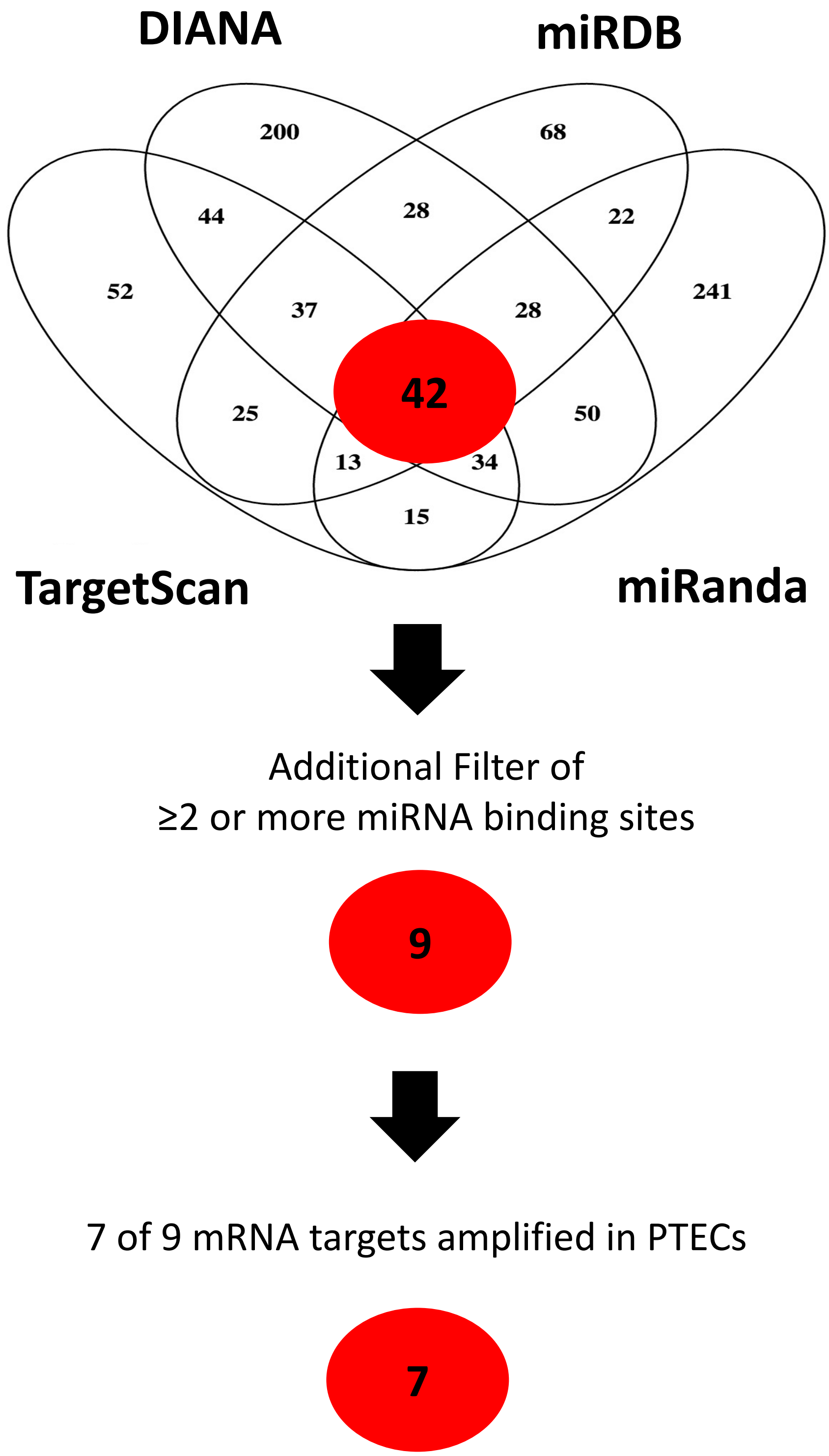

Supplementary Figure 2.
Identification of putative miR-141 mRNA targets by analysis *in silico*.
Combined data from analyses using Diana, miRDB, TargetScan and miRanda algorithms to predict miR-141 target mRNAs initially identified 42 target mRNAs. TargetScan highlighted 9 targets with 2 or more miR-141 seed sequence binding sites. RT-qPCR analysis detected expression of 7 of these targets in PTECs.

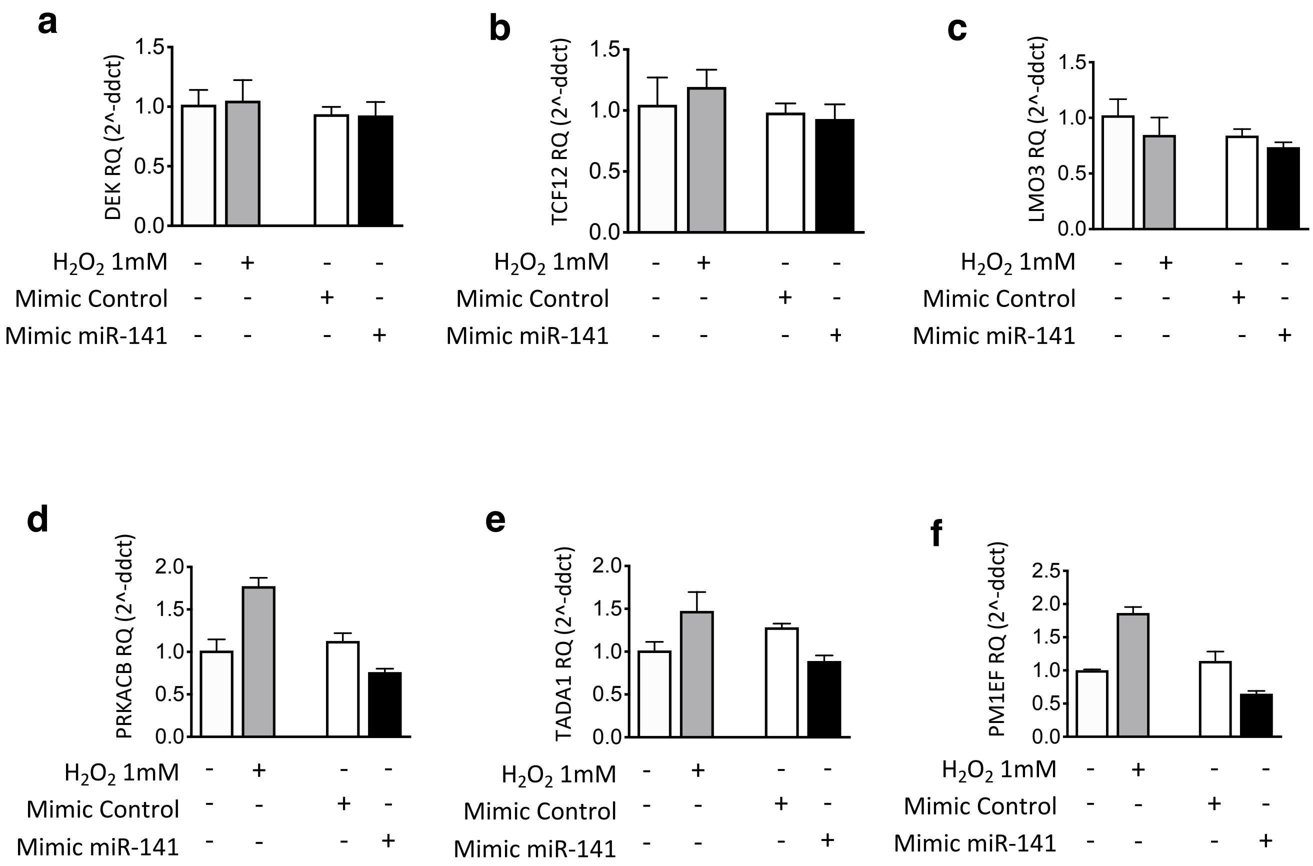

Supplementary Figure 3:

RT-qPCR analysis of six candidate target mRNAs of miR-141 determined *in silico*

(**a**-**g**) RT-qPCR analysis of six candidate miR-141 mRNA targets in PTECs following 1 mM H_20_2 treatment and/or forced miR-141 expression: (**a**) DEK, (**b**) TCF12 and (**c**) LMO3 showed no change, while (**d**) PRKACB, (**e**) TADA1 and (**f**) PM1EF were downregulated following forced miR-141 expression. Data are presented as standard deviation of triplicate assays (n = 1).

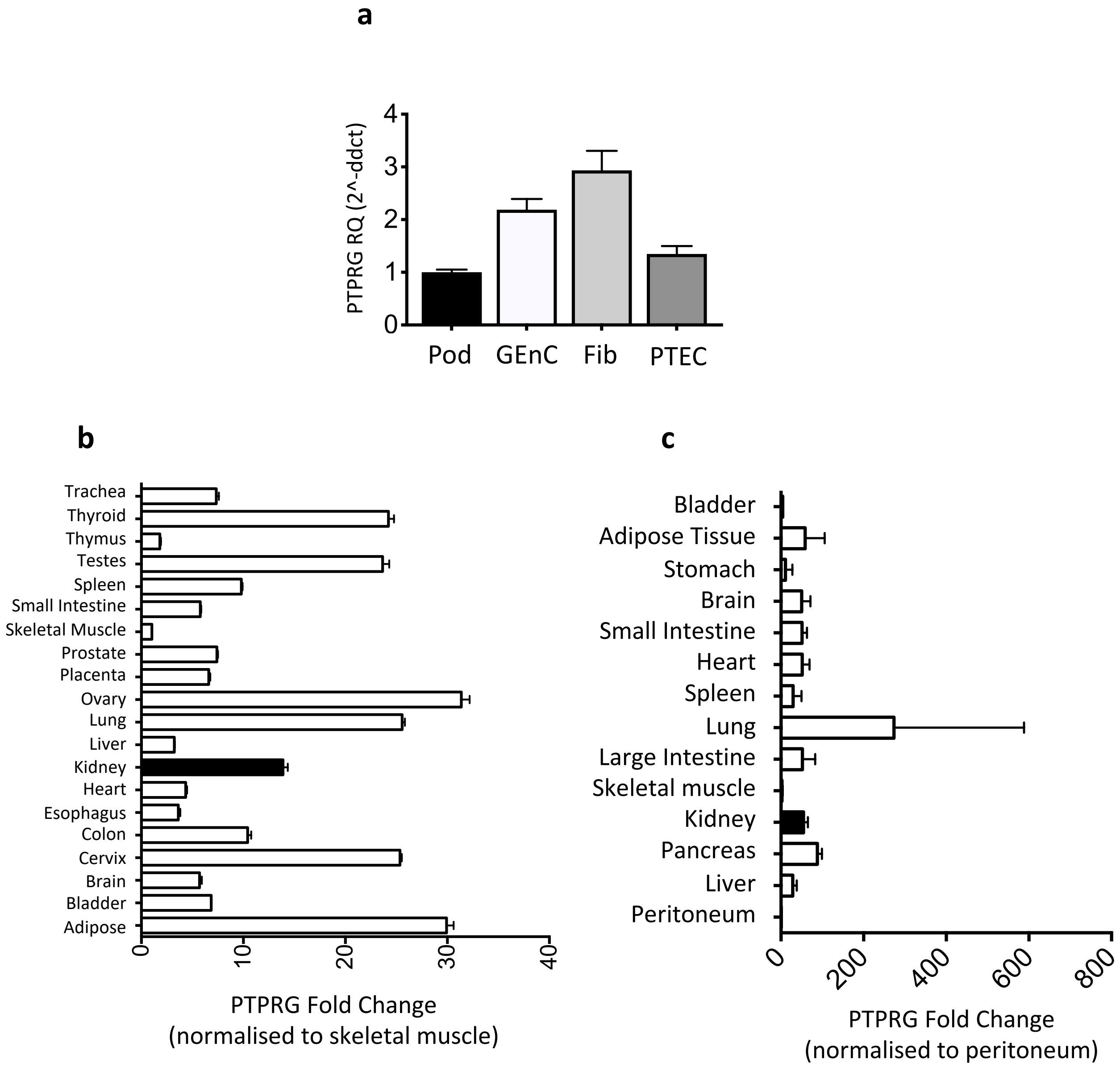

Supplementary Figure 4.

PTPRG expression in renal cell and in human and mouse global RNA libraries.

(a) RT-qPCR analysis detected PTPRG expression in podocytes (Pod), glomerular endothelial cells (GEnC), fibroblasts (Fib) and proximal tubule epithelial cells (PTEC). PTPRG expression was investigated in (b) human and (c) mouse global RNA libraries and expressed as fold change relative to skeletal muscle and peritoneum, respectively. (b) A 15-fold increase in PTPRG expression is observed in human kidney, and (c) a corresponding 100-fold increase is seen in mouse kidney.
